# Competing risk analysis of cardiovascular/cerebrovascular death in T1/2 kidney cancer: a SEER database analysis

**DOI:** 10.1186/s12885-020-07718-z

**Published:** 2021-01-05

**Authors:** Xiaofei Mo, Mingge Zhou, Hui Yan, Xueqin Chen, Yuetao Wang

**Affiliations:** 1 Department of Nuclear Medicine, the Third Affiliated Hospital of Soochow University, Changzhou, 213003 Jiangsu China; 2 Changzhou Key Laboratory of Molecular Imaging, Changzhou, 213003 Jiangsu China

**Keywords:** Kidney cancer, Cardiovascular and cerebrovascular death, SEER database, Competing risk analysis

## Abstract

**Background:**

Kidney cancer (KC) is associated with cardiovascular regulation disorder and easily leads to cardiovascular and cerebrovascular death (CCD), which is one of the major causes of death in patients with KC, especially those with T1/2 status. However, few studies have treated CCD as an independent outcome for analysis. We aimed to identify and evaluate the key factors associated with CCD in patients with T1/2 KC by competing risk analysis and compared these risk factors with those associated with kidney cancer-specific death (KCD) to offer some information for clinical management.

**Methods:**

A total of 45,117 patients diagnosed with first primary KC in T1/2 status were obtained from the Surveillance, Epidemiology, and End Results (SEER) database. All patients were divided into the CCD group (*n* = 3087), KCD group (*n* = 3212), other events group (*n* = 6312) or alive group (*n* = 32,506). Patients’ characteristics were estimated for their association with CCD or KCD by a competing risk model. Pearson’s correlation coefficient and variance inflation factor (VIF) were used to detect collinearity between variables. Factors significantly correlated with CCD or KCD were used to create forest plots to compare their differences.

**Results:**

The competing risk analysis showed that age at diagnosis, race, AJCC T/N status, radiation therapy, chemotherapy and scope of lymph node represented different relationships to CCD than to KCD. In detail, age at diagnosis (over 74/1–50: HR = 9.525, 95% CI: 8.049–11.273), race (white/black: HR = 1.475, 95% CI: 1.334–1.632), AJCC T status (T2/T1: HR = 0.847, 95% CI: 0.758–0.946) and chemotherapy (received/unreceived: HR = 0.574, 95% CI: 0.347–0.949) were correlated significantly with CCD; age at diagnosis (over 74/1–50: HR = 3.205, 95% CI: 2.814–3.650), AJCC T/N status (T2/T1: HR = 2.259, 95% CI: 2.081–2.451 and N1/N0:HR = 3.347, 95% CI: 2.698–4.152), radiation therapy (received/unreceived: HR = 2.552, 95% CI: 1.946–3.346), chemotherapy (received/unreceived: HR = 2.896, 95% CI: 2.342–3.581) and scope of lymph nodes (1–3 regional lymph nodes removed/none: HR = 1.378, 95% CI: 1.206–1.575) were correlated significantly with KCD.

**Conclusions:**

We found that age at diagnosis, race, AJCC T status and chemotherapy as the independent risk factors associated with CCD were different from those associated with KCD.

## Background

Kidney cancer (KC) is commonly diagnosed in older adults by chance. As one of the most common malignant cancers, KC represents the sixth most frequently diagnosed cancer in males and the 10th most frequently diagnosed cancer in females worldwide [1]. According to the World Health Organization, the newest data show that there are more than 140,000 KC-related deaths every year. Especially in Europe and North America, the lifetime risk for developing KC ranges from 1.3 to 1.8% [2]. Moreover, KC is ranked as the 13th leading cause of cancer death worldwide [3].

With respect to the pathology of KC, hypertension and increasing intracranial pressure are the common paraneoplastic disorders of KC [4]. These two paraneoplastic disorders are the main reasons for cardiovascular and cerebrovascular death (CCD) in KC patients.

CCD is one of the most prevalent causes of death in patients with KC, accounting for nearly 41.96% of all elderly patients, even in a long-term survival follow-up study [5]. In addition, nearly 70% of KC patients are diagnosed at American Joint Committee on Cancer Staging (AJCC) status T1/2 [6], which has a good prognosis. Considering that most KC patients have T1/2 status, it is necessary to analyse the risk factors related to CCD in these patients.

Unfortunately, many studies have attempted to quantitate the risks of kidney cancer-specific death (KCD) or overall survival [7–10], but few reports have analysed patients who face a high risk of CCD, so the precise clinical management of those patients is difficult to carry out.

We aimed to analyse the risk factors associated with CCD in patients with T1/2 KC, but CCD is one of the outcomes in KC. The outcomes of KC vary, and all of them are competing events. With respect to traditional survival analysis, all the other events are treated as censored events that may generate bias [11]. To analyse the event with competing events, Fine-Gray competing risk regression is a suitable method [12, 13]. Therefore, we carried out a competing risk model to identify risk factors for CCD and KCD in patients with T1/2 KC. We also used curved forest plots to compare those two kinds of factors.

## Methods

### Data source

The Surveillance, Epidemiology, and End Results (SEER) database (http://seer.cancer.gov/seerstat) covers approximately 30% of the US population and provides complete cancer patient data, including demographic, clinical information and follow-up data. This database is updated annually by the National Center for Health Statistics [14]. We chose 18 Registry Research Datasets (2000–2016, with additional treatment fields; November 2018 submitted) in the SEER database to identify cases for this study.

### Inclusion and exclusion criteria

We used cases that met the following criteria: (1) ICD-O-3 codes C64.9, C65.9 and C66.9, (2) KC is the first primary cancer, and (3) diagnosis between 2004 and 2015.

Cases were excluded according to the following criteria: (1) unknown demographic information, including race and marital status; (2) autopsy/death certification reports, which lack survival periods; (3) unknown clinical information, including American Joint Committee on Cancer (AJCC) stage, T/N/M status and tumour size; (4) cases without histological confirmation; (5) indefinite surgery of primary site information; and (6) patients diagnosed with KC at T3/4 status.

**Fig. 1:**
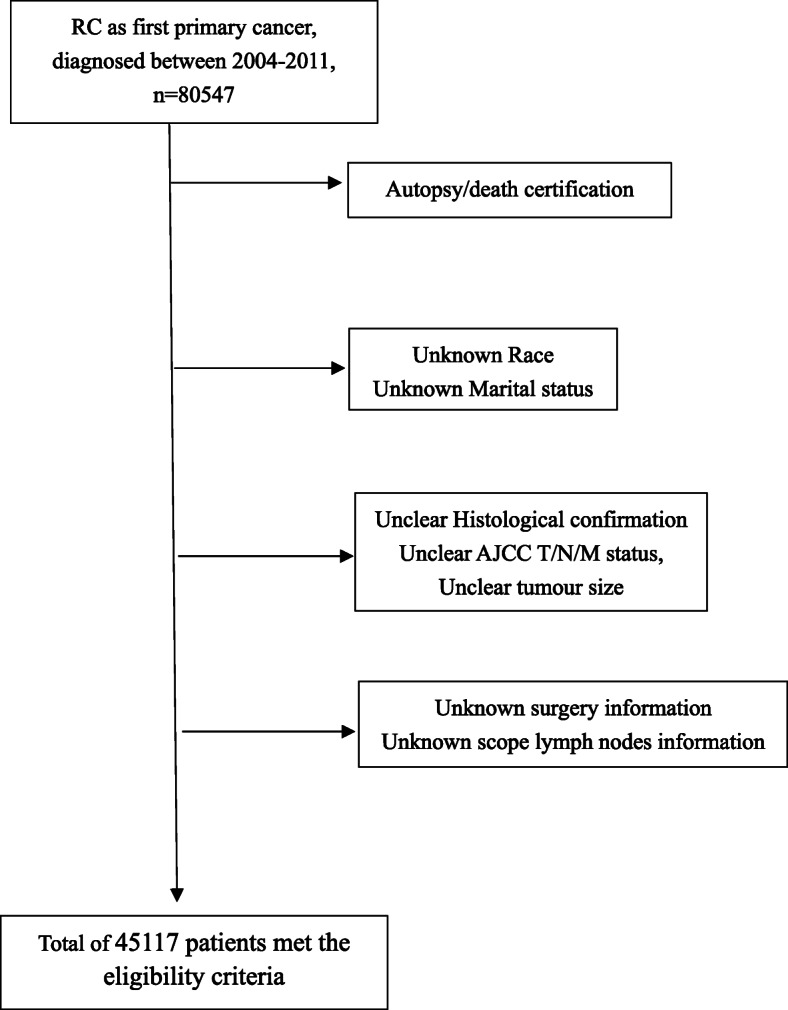
Flow chart of patient enrollment. Abbreviations: KC, kidney cancer; AJCC, American Joint Committee on Cancer

Then, the patients were divided into four groups according to their outcomes at the end of follow-up: (1) alive; (2) cardiovascular and cerebrovascular death (CCD), which includes diseases of the heart, cerebrovascular diseases and hypertension without heart disease; (3) kidney cancer-specific death (KCD); and (4) other events (OE), which includes developing a secondary primary cancer and other non-CCD/KCD causes of death. The inclusion and exclusion procedures mentioned above are shown in Fig. 1.

### Statistical analysis

First, we created a cumulative incidence function (CIF) plot, which showed the cumulative incidence of different outcomes in all included KC patients over time. The incidence rates of KCD and CCD after diagnosis at 36 months, 60 months and 120 months were calculated and compared.

Then, we used univariate and multivariate Fine-Gray’s competing risk models to analyse the hazard ratios (HRs) of each variable that significantly correlated with CCD and KCD [15]. Demographic and pathological characteristics and treatment information were considered in the analysis. After identifying the key variables, we curved forest plots to represent the significant risk factors and their HRs.

Variance inflation factor (VIF) values were calculated to measure the degree of multicollinearity among the variables [16, 17]. A VIF of > 5 indicates high correlation of the variables [18]. Pearson’s correlation coefficients were also calculated to detect collinearity among the variables. A correlation coefficient of < 0.7 between two independent variables was considered indicative of no multicollinearity [19].

The competing risk analyses and chi-square test in this study were conducted using R software (version: 3.6.3). Fine-Gray’s competing risk model was constructed with the R package cmprsk [20], and the forest plots were depicted with the R package forestplot. Multilinearity tests were operated with the R package car and corrplot.

To find the best cut-off values for continuous variables, such as age at diagnosis, the X-tile tool (Yale University, New Haven, Connecticut, USA) was used to estimate the most suitable cut-off points and has been widely used in many cancer-related studies [21, 22]. This tool uses an enumeration method to calculate the chi-square test results of different cut-off values according to the outcomes, and the minimum values represent the best cut-off points [23].

For competing risk analyses, differences in a set of HRs were evaluated by the chi-square test. For patients’ characteristics, differences in variables were evaluated by Fisher’s exact test (sample size ≤40) and the chi-square test (sample size > 40). A *p*-value of < 0.05 was considered statistically significant, and all tests were bilateral.

## Results

### Patients characteristics

As shown in Fig. 1, a total of 45,117 patients met the eligibility criteria, and among them, there were 32,506 (72.05%) patients in the alive group, 3087 (6.84%) in the CCD group, 3212 (7.12%) in the KCD group and 6312 (13.99%) in the OE group.

X-tile tool was used to find the optimal cut-off value of age at diagnosis as mentioned in statistical analysis section. The optimal cut-off values of age at diagnosis were 1–50, 51–58, 59–65, 66–73 and over 75.

As Table 1 shows, in the CCD group, the majority of patients were married (*n* = 1645, 53.29%), white (*n* = 2482, 80.40%), male (*n* = 1928, 62.46%) and diagnosed with KC at age ≥ 75 (*n* = 1400, 45.35%). Most patients were AJCC T1 (*n* = 2668, 86.43%), had N0 status (*n* = 3063, 99.22%) and had a tumour size > 3 cm (*n* = 2030, 65.76%). Regarding treatment, a minority of patients received radiation therapy (*n* = 23, 0.75%), chemotherapy (*n* = 16, 0.52%) and scope lymph nodes (*n* = 177, 5.73%), but most patients underwent surgery (*n* = 2570, 83.25%). Patient characteristics in the KCD group were similar to those in the CCD group. There were 1093 (34.03%) patients diagnosed at age > 75, 1890 (58.84%) married, 2670 (83.13%) white and 2035 (63.36%) male. There were 2039 (63.48%) patients with AJCC T1 status and 2992 (93.15%) with N0 status. Regarding patient treatment, 86 (2.68%) patients received radiation therapy, 164 (5.11%) received chemotherapy, 453 (14.10%) received scoping lymph nodes and 2606 (81.13%) underwent surgery. In the alive group, the patients’ characteristics were different from those of the KCD and CCD groups. There were 3280 (10.09%) patients diagnosed at age > 75, 22,042 (67.81%) married, 26,784 (82.40%) white and 19,568 males. There were 28,286 (87.02%) patients with AJCC T1 status and 32,389 (99.64%) with N0 status. Regarding patient treatment, 49 (0.15%) patients received radiation therapy, 139 (0.43%) received chemotherapy, 2252 (6.93%) received scoping lymph nodes, and 31,733 (97.62%) underwent surgery.

**Table 1:** Characteristics of the included patients with first primary kidney cancer

Characteristics	Group 1 (Alive)	Group 2 (CCD)	Group 3 (KCD)	Group 4 (OE)	*p*-value
Age at diagnosis					< 0.001
1–50	8366 (25.74%)	156 (5.05%)	316 (9.84%)	453 (7.18%)	
51–58	8512 (26.19%)	369 (11.95%)	577 (17.96%)	838 (13.28%)	
59–65	6804 (20.93%)	475 (15.39%)	591 (18.4%)	1091 (17.28%)	
66–73	5544 (17.06%)	687 (22.25%)	635 (19.77%)	1480 (23.45%)	
over 75	3280 (10.09%)	1400 (45.35%)	1093 (34.03%)	2450 (38.81%)	
Marital status					< 0.001
Sinlge/divorce/widow	10,464 (32.19%)	1442 (46.71%)	1322 (41.16%)	2833 (44.88%)	
Marry	22,042 (67.81%)	1645 (53.29%)	1890 (58.84%)	3479 (55.12%)	
Race					< 0.001
White	26,784 (82.40%)	2482 (80.40%)	2670 (83.13%)	5219 (0.10%)	
Asian/Pacific Islander	1701 (5.23%)	112 (3.63%)	163 (5.07%)	224 (3.55%)	
Black	3759 (11.56%)	474 (15.35%)	352 (10.96%)	809 (12.82%)	
American Indian/Alaska Native	262 (0.81%)	19 (0.62%)	27 (0.84%)	60 (0.95%)	
Sex					< 0.001
Female	12,938 (39.8%)	1159 (37.54%)	1177 (36.64%)	2385 (37.79%)	
Male	19,568 (60.2%)	1928 (62.46%)	2035 (63.36%)	3927 (62.21%)	
T (AJCC 6th)					< 0.001
I	28,286 (87.02%)	2668 (86.43%)	2039 (63.48%)	5398 (85.52%)	
II	4220 (12.98%)	419 (13.57%)	1173 (36.52%)	914 (14.48%)	
N (AJCC 6th)					< 0.001*
0	32,389 (99.64%)	3063 (99.22%)	2992 (93.15%)	6233 (98.75%)	
I	86 (0.26%)	21 (0.68%)	151 (4.7%)	64 (1.01%)	
II	31 (0.10%)	3 (0.10%)	69 (2.15%)	15 (0.24%)	
M (AJCC 6th)					< 0.001
0	32,506 (100%)	3087 (100%)	3212 (100%)	6312 (100%)	
I	0	0	0	0	
Tumour size					< 0.001
≤ 3 cm	13,379 (41.16%)	1057 (32.24%)	492 (15.32%)	2182 (34.57%)	
>3 cm	19,127 (58.84%)	2030 (65.76%)	2720 (84.68%)	4130 (65.43%)	
Radiation therapy					< 0.001*
No received	32,457 (99.85%)	3064 (99.25%)	3126 (97.32%)	6280 (99.49%)	
Received	49 (0.15%)	23 (0.75%)	86 (2.68%)	32 (0.51%)	
Chemotherapy					< 0.001*
No received	32,367 (99.57%)	3071 (99.48%)	3048 (94.89%)	6252 (99.05%)	
Received	139 (0.43%)	16 (0.52%)	164 (5.11%)	60 (0.95%)	
Scope of lymph node					< 0.001
None	30,254 (93.07%)	2910 (94.27%)	2759 (85.9%)	5908 (93.6%)	
1–3 regional lymph nodes removed	1466 (4.51%)	131 (4.24%)	299 (9.31%)	756 (4.40%)	
4 or more regional lymph nodes removed	786 (2.42%)	46 (1.49%)	154 (4.79%)	19,738 (2.00%)	
Surgey of primary site					< 0.001
No operation	773 (2.38%)	517 (16.75%)	606 (18.87%)	944 (14.96%)	
Local tumour destruction/excision	1555 (4.78%)	202 (6.54%)	101 (3.14%)	440 (6.97%)	
partly nephrectomy	13,940 (42.88%)	857 (27.76%)	611 (19.02%)	1809 (28.66%)	
total nephrectomy	16,238 (49.95%)	1511 (48.95%)	1894 (58.97%)	3119 (49.41%)	

^*^Differences were evaluated by Fisher’s exact test

*Abbreviations*: *CCD* cardiovascular and cerebrovascular death, *KCD* kidney cancer death, *OE* other events, *AJCC* American Joint Committee on Cancer

### Cumulative incidence function of CCD, KCD and OE

**Fig. 2:**
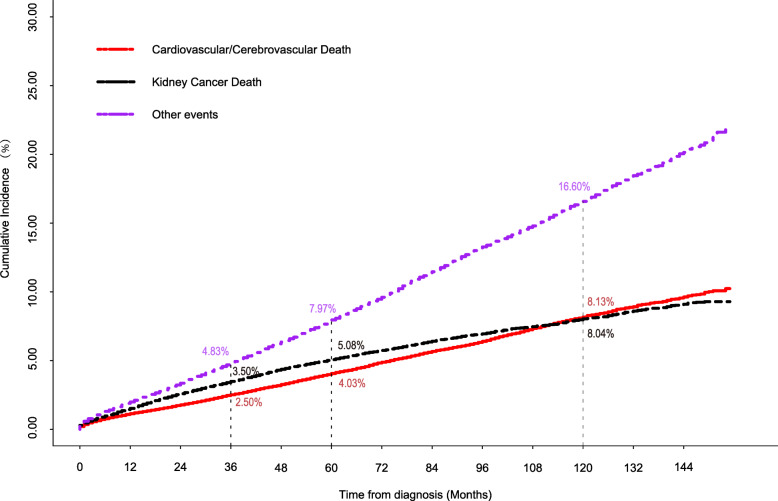
The cumulative function of CCD, KCD and OE in patients with T1/2 KC. Abbreviations: CCD, cardiovascular and cerebrovascular death; KCD: kidney cancer-specific death; OE, other events; KC, kidney cancer

We estimated the cumulative incidence ratios of each outcome via a competing risks model. As Fig. 2 shows, KCD and CCD represented almost the same cumulative incidence in patients after diagnosis with T1/2 KC. Thirty-six months after diagnosis, the cumulative incidences of CCD, KCD and OE were 2.50% (95% CI: 2.506–2.534%), 3.50% (95% CI: 3.486–3.514%) and 4.83% (95% CI: 4.826–4.834%), respectively. At 60 months after diagnosed, the cumulative incidences were 4.03% (95% CI: 4.016–4.044%), 5.08% (95% CI: 5.076–5.084%) and 7.97% (95% CI: 7.966–7.974%), respectively. At 120 months after diagnosed, the cumulative incidences were 8.13% (95% CI: 8.125–8.135%), 8.04% (95% CI: 8.035–8.045%) and 16.60% (95% CI: 16.595–16.605%), respectively.

### Univariate and multivariate analysis by Fine-Gray’s competing risk model

First, we used the univariate Fine-Gray’s competing risk model to analyse all factors listed above. In the CCD group, as Table 2 shows, except for American Indian/Alaska native status, sex and AJCC N status, all factors were significantly associated with CCD (*p* < 0.05). Then, we estimated those factors by multivariate Fine-Gray’s competing risk model and found that age at diagnosis, race, marital status, AJCC T status, chemotherapy and surgery of the primary site were significant risk factors for CCD. In detail, elderly patients had a higher risk of CCD, with HRs of 2.117 (95% CI: 1.756–2.552), 3.200 (95% CI: 2.672–3.832), 4.981 (95% CI: 4.187–5.925) and 9.525 (95% CI: 8.049–11.273) at age 51–58, 59–65, 66–73 and over 75 versus 1–50, respectively. Black patients faced a higher risk (black versus white: HR = 1.475, 95% CI: 1.334–1.632), but Asian/Pacific Islander patients had a lower risk of CCD (Asian/Pacific Islander versus white: HR = 0.826, 95% CI: 0.683–0.998). Married patients also showed a lower risk (married versus single/divorced/widowed patients: HR = 0.677, 95% CI: 0.628–0.730). For AJCC T status, T2 status had less risk of CCD than T1(HR = 0.847, 95% CI:0.758–0.946), but the tumour size> 3 cm group had higher risk (HR = 1.111, 95% CI:0.1.023–1.206); patients who received chemotherapy and surgery of the primary site also had reduced risk of CCD. In detail, the respective HRs were 0.574 (95% CI:0.347–0.949) for with chemotherapy versus without, and 0.631 (95% CI:0.532–0.747), 0.526 (95% CI:0.466–0.594), and 0.607 (95% CI:0.543–0.680) for with local tumour destruction/excision, with partial nephrectomy and with radical nephrectomy versus no operation.

**Table 2:** Univariate Fine-Gray’s competing risk model analysis for CCD and KCD in patients with first primary kidney cancer

CCD				KCD		
Characteristics	HR	95% CI for HR	*p-*value	HR	95% CI for HR	*p-*value
Age at diagnosis						
1–50	reference			reference		
51–58	2.131	1.760–2.560	< 0.001	1.650	1.440–1.890	< 0.001
59–65	3.180	2.651–3.809	< 0.001	1.944	1.693–2.217	< 0.001
66–73	4.973	4.182–5.908	< 0.001	2.240	1.966–2.563	< 0.001
over 74	10.713	9.079–12.641	< 0.001	4.071	3.594–4.621	< 0.001
Marital status						
Sinlge/divorce/widow	reference			reference		
Marry	0.608	0.566–0.652	< 0.001	0.771	0.719–0.827	< 0.001
Race						
White	reference			reference		
Asian/Pacific Islander	0.781	0.646–0.943	0.010	1.058	0.903–1.240	0.490
Black	1.366	1.238–1.507	< 0.001	0.922	0.825–1.030	0.150
American Indian/Alaska Native	0.775	0.495–1.214	0.270	1.036	0.708–1.511	0.860
Sex						
Female	reference			reference		
Male	1.070	0.992–1.15	0.082	1.113	1.030–1.191	0.005
T (AJCC 6th)						
1	reference			reference		
2	0.882	0.796–0.978	0.017	3.483	3.244–3.746	< 0.001
N (AJCC 6th)						
0	reference			reference		
1	0.954	0.616–1.48	0.830	9.821	8.237–11.703	< 0.001
2	0.369	0.118–1.15	0.086	14.498	11.151–18.808	< 0.001
Tumour size						
≤ 3 cm	reference			reference		
>3 cm	1.160	1.083–1.247	< 0.001	3.462	3.140–3.814	< 0.001
Chemotherapy						
No	reference			reference		
Yes	0.604	0.369–0.987	0.044	8.062	6.891–9.433	< 0.001
Radiation therapy						
No	reference			reference		
Yes	1.833	1.200–2.781	0.004	8.260	6.672–10.207	< 0.001
Scope lymph node						
None	reference			reference		
1–3 regional lymph nodes removed	0.838	0.704–0.999	0.048	2.122	1.892–2.388	< 0.001
4 or more regional lymph nodes removed	0.579	0.433–0.774	< 0.001	2.168	1.849–2.555	< 0.001
Surgey of primary site						
No operation	reference			reference		
Local tumour destruction/excision	0.134	0.107–0.166	< 0.001	0.179	0.145–0.221	< 0.001
partly nephrectomy	0.159	0.144–0.176	< 0.001	0.143	0.128–0.161	< 0.001
Radical nephrectomy	0.461	0.425–0.499	< 0.001	0.329	0.300–0.362	< 0.001

*Abbreviations*: *CCD* cardiovascular and cerebrovascular death, *KCD* kidney cancer death, *HR* hazard ratio, *CI* confidence interval, *AJCC* American Joint Committee on Cancer

In the KCD group, as Table 2 shows, age at diagnosis, marital status, sex, AJCC TN status, tumour size, radiation therapy, chemotherapy, scope lymph nodes and surgery of the primary site were significantly associated with this outcome by the univariate competing risk model. Then, we estimated these factors by a multivariate competing risk model, and these factors were still significantly associated with KCD. In detail, elderly patients still showed a higher risk of KCD, and the respective HRs were 1.578 (95% CI: 1.376–1.810), 1.933 (95% CI: 1.688–2.215), 2.176 (95% CI: 1.899–2.494) and 3.205 (95% CI: 2.814–3.150) at ages 51–58, 59–65, 66–73 and over 75 versus 1–50, respectively. Married patients showed a lower risk of KCD (married versus single/divorced/widowed: HR = 0.867, 95% CI: 804–0.935). For AJCC T status, T2 status had higher risks of KCD than T1 (HR = 2.259, 95% CI:2.081–2.451), and advanced N status had higher risks of KCD; the respective HRs were 3.347 (95% CI:2.698–4.152) and 4.004 (95% CI: 2.837–5.650) for N1 and N2 versus N0 status; the tumour size> 3 cm group had higher risk (HR = 2.319, 95% CI:2.086–2.579). Patients who received chemotherapy, radiation therapy and scope of lymph nodes still had high risks of KCD, and the respective HRs were 2.896 (95% CI: 2.342–3.581) for with chemotherapy versus without; 2.552 (95% CI: 1.946–3.346) for with radiation therapy versus without, 1.378 (95% CI: 1.206–1.575) for with 1–3 regional lymph nodes removed, and 1.230 (95% CI: 1.022–1.480) for 4 or more regional lymph nodes removed versus no scoping of lymph nodes. Patients who underwent surgery at the primary site faced lower risks of KCD, with HRs of 0.356 (95% CI: 0.286–0.443), 0.275 (95% CI: 0.242–0.312) and 0.393 (95% CI: 0.352–0.438) for local tumour destruction/excision, partial nephrectomy and radical nephrectomy, respectively, versus no operation.

**Fig. 3:**
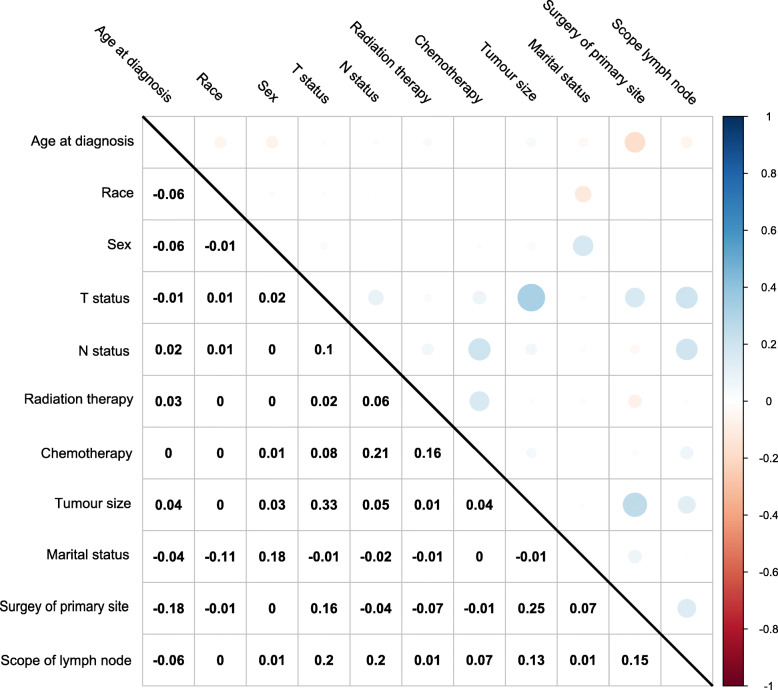
Pearson’s correlation coefficients between pairs of variables

**Fig. 4:**
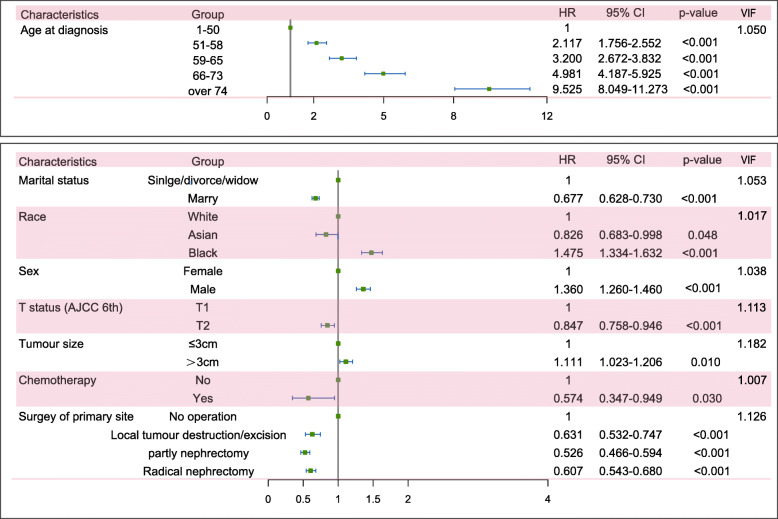
Forest plot of the risk factors associated with CCD. Abbreviations: CCD, cardiovascular and cerebrovascular death; VIF: variance inflation factor; AJCC: American Joint Committee on Cancer; HR: hazard ratio; CI: confidence interval

**Fig. 5:**
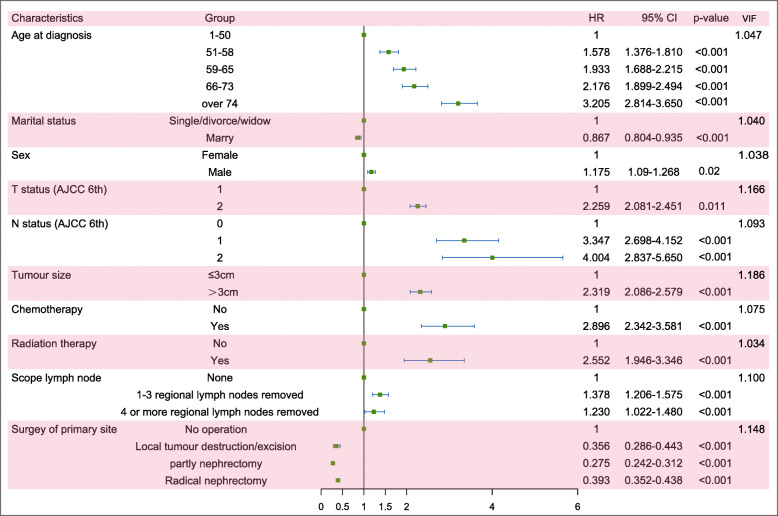
Forest plot of the risk factors associated with KCD. Abbreviations: KCD: kidney cancer-specific death; VIF: variance inflation factor; AJCC: American Joint Committee on Cancer; HR: hazard ratio; CI: confidence interval

All the correlation coefficients between pairs of variables were < 0.7 and the VIF values were close to 1, indicating no collinearity among the independent variables (Figs. 3, 4 and 5).

### Forest plots of the risk factors and hazard ratios of CCD and KCD

We summarized the multivariate competing risk analysis and the VIF test results of CCD and KCD in patients with T1/2 kidney cancer and curved forest plots to visualize these results, which are shown in Figs. 4 and 5. Comparing these results, we found that there were some differences in the risk factors that were significantly associated with CCD or KCD. For CCD, age was the largest risk factor with the highest HRs, and race was also associated with CCD, but the N status and scope of lymph nodes did not show a relationship with CCD. However, for KCD, age did not represent comparatively high HRs to KCD, and race was not associated with KCD. Moreover, N status was the largest risk factor associated with KCD, and T status showed opposite HRs to KCD compared with CCD. Radiation therapy, chemotherapy and scope of lymph nodes also showed different HRs to CCD and KCD, but surgery of the primary site had similar HRs.

## Discussion

In this study, we aimed to analyse CCD in patients with T1/2 KC based on the SEER database. A total of 45,117 patients with first primary T1/2 KC were screened to curve the CIF of CCD and KCD, and the competing risk model was used to estimate the risk factors associated with CCD and KCD. Age at diagnosis, marital status, race, T status, tumour size, chemotherapy and surgery of the primary site were significantly associated with CCD; age at diagnosis, marital status, sex, T/N status, tumour size, chemotherapy, radiation therapy, scope of lymph node and surgery of the primary site were significantly associated with KCD. The HRs of these factors were estimated and then visualized by forest plots to compare these risk factors.

Although many previous studies on survival analyses for patients with KC have been performed, including assessment of postoperative overall survival, early-stage KC-specific survival, metastatic KC-specific survival and so on [8, 9, 24, 25], there is still a lack of CCD-related analysis in patients with KC, especially in T1/2 status, which occupies nearly 70% of KC diagnoses and has a relatively high survival probability. Moreover, most of the previous studies analysed overall survival by treating all outcomes as one, which might not be suitable to identify KC patients with different risks of various outcomes. In the present study, we used a competing risk model to analyse CCD and KCD in patients with T1/2 KC, as the best mechanism to obtain more precise results and help clinical management make decisions.

CCD is one of the main outcomes in patients with cancer, and the incidence rate of CCD is greater than that in cancer-free people. In the present work, the CIF we plotted showed many characteristics of T1/2 KC. First, the incidence rate of CCD was similar to that of KCD from the beginning of diagnosis to 10 years later. This feature indicated that CCD was as important as KCD in T1/2 KC. Second, the cumulative incidence rate of CCD increased smoothly, which indicated that after diagnosis with KC, the risk of CCD did not change year by year. This result was different from other cancer-specific CCDs reported previously by Fang [26]. They used a large cohort study to conclude that the incidence rate of cardiovascular death after diagnosis was highest in the first year and decreased year by year. This feature indicated that the management of patients with KC required a long period to prevent cardiovascular events.

Regarding the risk factors for CCD and KCD, in the present study, we found that age was the predominant risk factor associated with CCD and that elderly patients faced a higher hazard risk of CCD, but this factor did not show such a predominant effect on KCD, which was also proven by other previous studies [27]. Black patients faced a higher risk of CCD than other races, and Asians faced the lowest risk of CCD, but race was not a significant factor associated with KC-specific death, as also reported by Kun-Chi [28] et al. and Yuan Z et al. [29]. Marital status showed a similar hazard risk of CCD and KCD: married patients faced a lower risk than those who were single/divorced/widowed.

Regarding the pathological characteristics of patients, those with KC who stayed in T2 status showed a lower risk of CCD but a higher risk of KCD than those with T1 status. N status was the predominant factor associated with KCD but did not show a significant association with CCD. Advanced AJCC status represents giant tumour size, distant metastasis and lymph node invasion, which are significantly associated with cancer-specific death and the development of secondary cancer [30], but the results in the present study showed that AJCC status might not be suitable for predicting CCD in KC. Unfortunately, there is a lack of other related research about AJCC status with CCD in KC to prove this result, so this conclusion should be verified by further studies.

For surgically treated KC patients, those who underwent tumour excision surgery or nephrectomy had lower risks of CCD in our study. This result indicated that surgery could reduce the risk of CCD in KC patients. However, in terms of different surgery types, there is no consensus about long-term cardiovascular events after surgery. Some reports suggested that KC patients with radical nephrectomy had a higher risk of cardiovascular events than those with partial nephrectomy, which was similar to our results. Huang et al. analysed SEER Medicare and concluded that nearly 20% of cardiovascular events increased after KC patients were treated with radical nephrectomy compared with nephron-sparing surgery [31]. Umberto et al. reported that nephron-sparing surgery showed an independent protective effect on hypertensive KC patients without preoperative cardiovascular disease but not on other major cardiovascular events [32]. One of the possible pathophysiological mechanisms of this finding is that the acute loss of half of the nephrons may induce a compensatory function of the remaining kidney that increases arterial blood inflow by activating the renin-angiotensin-aldosterone system [33, 34]. Although there have been many reports showing that radical nephrectomy generates more risk of cardiovascular events for KC patients than nephron-sparing surgery [35], the surgical plan should be made based on the individual patient benefit, and the risk of cardiovascular events after surgery should be considered. It is also necessary to pay attention to postoperative care to prevent acute hypertension. Additionally, the relationship between different surgery types and postoperative cardiovascular events in KC patients need to be determined, which could help clinicians make optimal treatment decisions.

For radiation therapy and chemotherapy, neither of these therapy methods were popular in KC therapy, so few patients (nearly 1%) with KC received these therapy methods. In our study, chemotherapy decreased the risk of CCD but increased the risk of KCD in KC patients, which was contrary to common sense due to the bias existing in our study. One possible explanation is that the patients who received chemotherapy were considered to have a poor prognosis, but those who did not receive chemotherapy were considered to have a good prognosis. Radiation therapy and chemotherapy were confirmed to be useful for KC treatment in many respects, such as reducing the risk of local recurrence [36], delaying the metastasis of cancer [37]and so on [38], but there were rare studies about the effect of radiation therapy and chemotherapy on CCD in KC patients. Further studies are expected to be conducted in the clinic and laboratory to provide more information on the relationship between these two therapeutic methods and cardiovascular/cerebrovascular events in KC patients.

There are several limitations existing in this study. First, our analysis quantifies the hazard risk of many variables associated with CCD and KCD but does not take patients’ comorbidities into account due to the limitations of SEER. Nevertheless, the results in this study are still meaningful and should be improved. Second, because the demographic and clinical information provided by the SEER database is not complete, more than 20,000 individuals were excluded, which may lead to some selection bias. Third, retrospective studies have intrinsic limitations, such as selection bias and experimental bias. Although several limitations exist in this study, the analysis in the present study was still meaningful and can offer some information for clinical management.

## Conclusions

We performed competing risk analyses of CCD and KCD in patients with first primary T1/2 kidney cancer based on the SEER database. Significant contributing factors for CCD and KCD were identified, and their hazard ratios were calculated. Age at diagnosis, race, marital status, sex, AJCC T status, chemotherapy and surgery of the primary site were associated with CCD. Among these factors, age at diagnosis, race, AJCC TN status and chemotherapy represented different relationships to CCD than KCD. Further studies of CCD in patients with KC are expected to establish a practical prognostic model for clinical use.

## Data Availability

The datasets analyzed during the current study are available in the SEER repository (https://seer.cancer.gov/). The databases are public access.

## Abbreviations

KC: Kidney cancer
CCD: Cardiovascular and cerebrovascular death
KCD: Kidney cancer-specific death
OE: Other events
SEER: Surveillance, Epidemiology, and End Results
CIF: Cumulative incidence function
AJCC: American Joint Committee on Cancer
HR: Hazard ratio
CI: Confidence interval
